# Overexpression of cystatin C in synovium does not reduce synovitis or cartilage degradation in established osteoarthritis

**DOI:** 10.1186/s13075-015-0519-3

**Published:** 2015-01-16

**Authors:** Sirkka Kyostio-Moore, Susan Piraino, Patricia Berthelette, Nance Moran, Joseph Serriello, Alison Bendele, Cathleen Sookdeo, Bindu Nambiar, Patty Ewing, Donna Armentano, Gloria L Matthews

**Affiliations:** Gene Therapy, Genzyme, a Sanofi Company, 49 New York Avenue, Framingham, MA USA; Orthopaedic Research, Genzyme, a Sanofi Company, 49 New York Avenue, Framingham, MA USA; Pathology, Genzyme, a Sanofi Company, 5 Mountain Road, Framingham, MA USA; Bolder BioPATH, 5541 Central Avenue, Boulder, CO USA

## Abstract

**Introduction:**

Cathepsin K (catK) expression is increased in cartilage, bone and synovium during osteoarthritis (OA). To study the role of catK expression and elevated cathepsin activity in the synovium on cartilage destruction in established OA, we overexpressed cystatin C (cysC), a natural cysteine protease inhibitor, in the synovium of rabbit OA joints.

**Methods:**

The ability of cysC to inhibit activity of cathepsins in rabbit OA synovium lysates was tested *in vitro* using protease activity assay. *In vivo*, the tissue localization of recombinant adeno-associated virus (rAAV) with *LacZ* gene after intra-articular injection was determined by β-galactosidase staining of rabbit joints 4 weeks later. To inhibit cathepsin activity in the synovium, a rAAV2-encoding cysC was delivered intra-articularly into rabbit joints 4 weeks after OA was induced by anterior cruciate ligament transection (ACLT). Seven weeks postinjection, endogenous catK and cysC levels as well as the vector-derived cysC expression in the synovium of normal and OA joints were examined by RNA quantification. Synovial cathepsin activity and catK, catB and catL protein levels were determined by activity and Western blot analyses, respectively. Synovitis and cartilage degradation were evaluated by histopathological scoring.

**Results:**

*In vitro*, the ability of cysC to efficiently inhibit activity of purified catK and OA-induced cathepsins in rabbit synovial lysates was demonstrated. *In vivo*, the intra-articular delivery of rAAV2/LacZ showed transduction of mostly synovium. Induction of OA in rabbit joints resulted in fourfold increase in catK mRNA compared to sham controls while no change was detected in endogenous cysC mRNA levels in the synovium. Protein levels for catK, catB and catL were also increased in the synovium with a concomitant fourfold increase in cathepsin activity. Joints treated with rAAV2/cysC showed both detection of vector genomes and vector-derived cysC transcripts in the synovium. Production of functional cysC by the vector was demonstrated by complete block of cathepsin activity in the synovium. However, this did not decrease synovitis, bone sclerosis or progression of cartilage degradation.

**Conclusions:**

Increased production of natural cathepsin inhibitor, cysC, in OA synovium does not alleviate synovitis or cartilage pathology during a preexisting OA.

## Introduction

The hallmark of osteoarthritis (OA), cartilage degradation, is caused by an imbalance of anabolic and catabolic pathways with a shift toward increased catabolic pathways that include upregulation of multiple proteases in the OA joint. These include matrix metalloproteases (MMPs), aggrecanases (ADAMs, ADAM-TS) and cathepsins, all of which are thought to play a role in articular cartilage degradation [1-5]. Of these, cathepsins including cathepsin K (catK) are ubiquitous cysteine-type endopeptidases that are mostly located in the lysosomes but are also secreted into the extracellular milieu [6-10]. Besides contributing to bone remodeling by secretion by osteoaclasts, the role of catK in cartilage degradation is well-supported by its ability to cleave both the triple-helical structure of type II collagen and aggrecan, and its elevation in cartilage and synovium both in humans and animal models of OA [3,7-13]. In naturally occurring equine OA, increased levels of catK and its degradation fragments in cartilage correlate to the severity of the disease particularly at the later stages of OA [14]. Furthermore, upregulation of catK in transgenic mice resulted in synovitis and cartilage degradation while OA was delayed in catK knockout (KO) mice indicating that catK can contribute to OA pathology [15,16].

CatK as a therapeutic target for OA is supported by reports demonstrating that treatment with catK inhibitors provided systemically at the early stages of the disease reduced the severity of cartilage pathology in surgically-induced rabbit and canine OA models and pain in a guinea pig model of spontaneous OA [17-19]. It is, however, unclear whether inhibitors in these studies preferentially target catK activity in subchondral bone or whether catK expression in the synovium, either locally or via secretion into synovial fluid, contributes to cartilage pathology. In a transgenic mouse model, catK upregulation augmented synovial inflammation and cartilage degradation [15]. However, in a rabbit OA model downregulation of catK in the synovium at the time of disease initiation increased cartilage pathology suggesting a protective role [20]. Factors produced by synovium such as proteases and inflammatory mediators may contribute to cartilage degradation and in recent OA clinical trials, pathology of synovium was shown to be predictive for both OA pain and cartilage degradation [21,22].

Our objectives were to study the role of catK expression and cathepsin activity in the synovium on cartilage degradation during a preexisting disease. We examined this by using cystatin C (cysC) [8,23,24], a natural cathepsin inhibitor, with the following goals: (1) to test inhibition of catK and cathepsin activity in rabbit synovium by cysC *in vitro*, (2) to quantitate the endogenous catK and cysC mRNA levels and well as catK, catB and catL protein levels in the synovium in a rabbit OA model *in vivo* and (3) to inhibit cathepsin activity in the rabbit OA synovium by overexpression of cysC mediated by the local delivery of cysC cDNA with recombinant adeno-associated virus (rAAV).

## Methods

### Cathepsin activity assay

Purified pro-catK (human recombinant), catS (human recombinant) and catB and catH (both from human liver) (Calbiochem/EMD Millipore, Billerica MA, USA) were used. Pro-catK was activated at pH 4.0 for 60 min at 25°C in NaOAc buffer containing 5 mM DTT and 0.5 mM EDTA. Cathepsins (0.03 nM to 60 nM) were assayed in pH 5.5 MES buffer using a fluorogenic substrate Z-Phe-Arg-amido-4-methylcoumarin (10 to 50 μM; Z-Phe-Arg-AMC; Calbiochem) [8,9,23] in a 96-well plate. The plates were incubated at 25°C followed by measurement of fluorescence at Ex/Em = 355/460 nm at various time points. Data were graphed as relative fluorescence units (RFU) and EC_50_ values were determined by DeltaGraph software (Red Rocks Software, Salt Lake City UT, USA). Inhibition by cysC (human or mouse; R&D Systems, Minneapolis, MN, USA), trans-epoxysuccinyl-L-leucylamido-(4-guanidino)butane) (E-64; Sigma-Aldrich, St Louis, MO, USA) or CA-074 (catB-specific inhibitor; Calbiochem) was calculated as percentage of activity compared to no inhibitor present.

To quantitate cathepsin activity in rabbit synovium, tissues were homogenized using 1mm zirconia beads (Biospec Products Inc, Bartlesville, OK, USA) in Tper buffer (20 μl/mg of tissue; Thermo Fisher Scientific, Waltham, MA, USA) and serine protease inhibitor cocktail for 30 s followed by cooling in ice for 30 s and repeated five times. Homogenates were centrifuged and supernatants (30 to 50 ug total protein/sample) were assayed for cathepsin activity using 50 μM substrate and pH 5.5 as described above (without low pH activation). Relative activity was expressed as RFUs or values were normalized by total protein content measured by BCA assay (Thermo Fisher Scientific).

### rAAV vector production

LacZ expression cassette contained a human cytomegalovirus (CMV) promoter, *Escherichia coli LacZ* gene and a bovine growth hormone (BGH) polyA. CysC vector contained a cDNA-encoding mouse cysC (GenScript Inc, Piscataway, NJ, USA), a CMV enhancer-chicken β-actin promoter and BGH polyA. A negative control, an empty vector (EV), contained CMV promoter and BGH polyA but no transgene. All expression cassettes were cloned into a plasmid pAAVSP70 containing AAV type 2 inverted terminal repeats [25]. rAAV1 and rAAV2 vectors were created by cross-packaging of AAV genomes into AAV1 or AAV2 serotype capsids, respectively [26]. rAAV was generated by triple transfection method and purified using iodixanol columns [27]. Vector titers were quantitated by TaqMan real-time PCR (RT-PCR) using primers and probe specific to the BGH polyA and values expressed as DNAse-resistant particles (drp) per ml [27].

### *In vitro* cell culture experiments and protein analyses

All enzymes and tissue culture reagents were purchased from Invitrogen (Carlsbad, CA, USA). Primary chondrocytes and synoviocytes were dissected from rabbit knee joints and digested with 0.5 mg/ml collagenase at 37°C overnight in DMEM followed by culture in DMEM, 10% fetal bovine serum (FBS), 100 IU/ml penicillin and 100 μg/ml streptomycin in 5% CO_2_ incubator at 37°C. For catK secretion studies, cells (3 × 10^5^ cells/well) were plated on 6-well dishes using DMEM media with high glucose, 10% FBS and 50 μg/ml gentamicin sulfate. Twelve hours later, interleukin 1 beta (IL-1β) (R&D Systems) was added at 2 ng/ml to some wells. Conditioned media was harvested 48 h later and concentrated sixfold by centrifugation (Amicon Ultra-5 centrifuge columns (EMD Millipore)) followed by protein analysis.

For rAAV infection, synoviocytes and chondrocytes were plated as described above and 1 × 10^5^ drp/cell of rAAV vectors were incubated for 5 h in the presence of adenovirus temperature-sensitive 149 helper virus (Adts149; multiplicity of infection of 10) followed by media change into media with 2.5% FBS. Culture media was collected 72 h later.

Culture media from *in vitro* experiments and rabbit synovial lysates were analyzed for protein detection by Western blotting. Samples were run on a 4 to 12% NuPAGE gel (Invitrogen) in MES-reducing buffer and transferred to a nitrocellulose membrane. The membrane was blocked with 0.15% I-Block (Life Technologies, Grand Island, NY, USA) in phosphate-buffered saline (PBS) and 0.05% Tween for 2 h. Cathepsins were detected using mouse monoclonal antibodies to catK (C8243; Sigma-Aldrich), catB (C6243; Sigma-Aldrich) and catL (ab6314; Abcam, Cambridge, USA) followed by goat anti-mouse immunoglobulin G (IgG)-horseradish peroxidase (HRP) conjugate. CysC was detected using goat anti-mouse cysC antibody (R&D Systems) followed by rabbit anti-goat-HRP. Beta-actin was detected with rabbit antibody-HRP conjugate (Cell Signaling Technology, Danvers, MA, USA). Purified human catK (Calbiochem or Enzo Life Sciences, Farmingdale, NY, USA), catB and catL (Calbiochem) and mouse cysC (R&D Systems) were used as positive controls. Detection was performed using ECL detection kit (Thermo Fisher Scientific). The density of bands was quantitated using ImageJ [28] and values were normalized to those obtained for sham animals.

### Animal experiments

All protocols were approved by the Genzyme Institutional Animal Care and Use Committee and conducted in accordance with humane guidelines for animal care and use. Male New Zealand White rabbits (Millbrook Breeding Labs, Amherst, MA, USA) at 8 to 12 months of age (3.5 to 5.0 kg) were used for animal studies. For rAAV serotype studies in normal joints, rAAV1 or rAAV2 vectors containing *LacZ* gene or rAAV2/EV (5 × 10^11^ drp/700 μl/joint; n = 3/treatment) were administered into the intra-articular space of the knee joint using a 25-gauge needle and the rabbits were euthanized 4 weeks later for analysis of β-galactosidase activity.

For OA studies, 45 rabbits underwent anterior cruciate ligament transection (ACLT) surgery as described previously [29]. A sham control (contralateral limb) consisted of an incision and closure of the skin. Animals then received appropriate postoperative care and were allowed to free roam after an initial recovery of 5 days post surgery. Four weeks post surgery, ACLT-treated joints were divided into three treatment groups of fifteen animals each. To each group, test articles consisting of PBS, rAAV2/EV (vector with no transgene) or rAAV2/mCysC (vector encoding mouse cysC) were administered by intra-articular route and all opposing sham knee joints received PBS (700 μl/joint for all treatments). Animals were sacrificed at 7 weeks postinjection. For some experiments, animals were euthanized at 4 weeks post ACLT for baseline data. Synovium was collected for RNA, DNA, cathepsin activity (around femoral condyle) and histology analyses (fat pad). Tibias and femurs were fixed in 4% paraformaldehyde (PFA), decalcified in 10% formic acid for 3 days and then cut into two frontal halves (anterior and posterior). All histology samples were embedded into paraffin and 8 μM sections were cut and stained with toluidine blue (cartilage) or with hematoxylin and eosin (H&E) (synovium).

### Joint staining for β-galactosidase activity

Skin was removed from the rabbit knee joints and the patellar ligament was cut to expose the joint space. The joints were fixed with 2% PFA at 4°C for 1 h followed by three rinses in PBS. Joints were incubated in 1.2 mg/ml solution of X-gal (Invitrogen) in PBS, pH 7.4 containing 5.2 mM potassium ferricyanide, 5.0 mM potassium ferrocyanide, 1mM magnesium chloride, 0.01% sodium deoxycholic acid (sodium salt) and 0.02% Igepal CA-630 (chemicals from Sigma-Aldrich) overnight at 37°C. After PBS rinse, synovium was collected from the fat pad, around the patella, suprapatellar pouch and femur and fixed in 10% normal buffered formalin (NBF) for 48 h. Tibiofemoral joints were fixed in 10% NBF for 72 h, decalcified in EDTA and sectioned coronally. Tissues were embedded in paraffin, sectioned at 5 microns and stained with H&E and nuclear fast red (NFR). NFR-counterstained sections were evaluated for location of β-gal positive cells by a board-certified pathologist (PE).

### Macroscopic and histological assessment of joints

Cartilage degradation in rabbit joints was assessed by two methods. First, macroscopic evaluation was performed for tibial and femoral cartilage surfaces stained with India ink. The area of stained surface (consisting of minor fibrillation to total loss of cartilage) in six areas (tibial plateaus, femoral epicondyles and condyles; each evaluated on medial and lateral sides) was measured with a ruler under a dissection microscope. Additionally, the most severe damage in each area was scored according to the 0 to 4 grading system described by Chang *et al*. [30]. The total score was defined as the size of the stained area multiplied by the severity score. Second, histological evaluation of joint sections from separated tibias and femurs (both cut into anterior and posterior halves) were performed in a blinded manner by a board-certified veterinary pathologist (AP) as described by Gerwin *et al*. [31]. Briefly, toluidine blue-stained 8 μM sections were examined for cartilage degradation (proteoglycan/chondrocyte loss, collagen damage and cartilage atrophy) and scored using a 0 to 5 grading system (Table 1). Additionally, the total area of cartilage affected by any type of degradation (cell or proteoglycan loss, collagen damage) in tibia and femur (both medial and lateral sides) was measured as cartilage degradation width. The degradation scores and widths were then summed up to obtain total cartilage degeneration scores. Synovial sections stained with H&E were used to score for synovial inflammation based on accumulation of mononuclear inflammatory cells (macrophages and lymphocytes) in the synovial lining as described in Table 2. Lastly, bone morphology was analyzed by scoring for osteophyte formation and bone sclerosis. For the former, the largest osteophyte in each compartment (medial and lateral) was scored according to its size based on measurement with an ocular micrometer (Table 3). For bone sclerosis, cartilage sections from tibia and femur were scored based on subchondral or epiphyseal trabecular bone thickness by comparing the medial side to lateral side as well as to sections from sham-treated animals (Table 4).

**Table 1:** **Descriptions for cartilage degeneration scoring**

**Score**	**Description**
0	No degeneration
1	Minimal degeneration, within the zone 3-10% of the matrix appears nonviable as a result of significant chondrocyte loss (greater than 50% of normal cell density lost in affected areas). Proteoglycan loss is usually present in these areas of cell loss and collagen matrix loss may be present
2	Mild degeneration, within the zone 11-25% of the matrix appears nonviable as a result of significant chondrocyte loss (greater than 50% of normal cell density lost in affected areas). Proteoglycan loss is usually present in these areas of cell loss and collagen matrix loss may be present
3	Moderate degeneration, within the zone 26-50% of the matrix appears nonviable as a result of significant chondrocyte loss (greater than 50% of normal cell density lost in affected areas). Proteoglycan loss is usually present in these areas of cell loss and collagen matrix loss may be present
4	Marked degeneration, within the zone 51-75% of the matrix appears nonviable as a result of significant chondrocyte loss (greater than 50% of normal cell density lost in affected areas). Proteoglycan loss is usually present in these areas of cell loss and collagen matrix loss may be present
5	Severe degeneration, within the zone 76-100% of the matrix appears nonviable as a result of significant chondrocyte loss (greater than 50% of normal cell density lost in affected areas). Proteoglycan loss is usually present in these areas of cell loss and collagen matrix loss may be present

**Table 2:** **Descriptions for synovial pathology scoring**

**Score**	**Description**
0	No pathological changes
1	Minimal multifocal small (no measurable aggregates) subsynovial accumulations of inflammatory cells
2	Diffuse small (no measurable aggregates) subsynovial accumulations of inflammatory cells
3	Diffuse small subsynovial accumulations of inflammatory cells + 1–3 aggregates measuring at least 100 µM in diameter
4	Diffuse small subsynovial accumulations of inflammatory cells + >3 aggregates measuring at least 100 µM in diameter
5	Diffuse severe inflammation with numerous aggregates

**Table 3:** **Descriptions for osteophyte scoring**

**Score**	**Description**
0	None
1	Small (200–1000 µM)
2	Medium (1001–1500 µM)
3	Large (1501–2000 µM)
4	Very large (2001–2500 µM)
5	Greater than 2500 µM

**Table 4:** **Descriptions for bone sclerosis scoring**

**Score**	**Description**
0	Normal, no observable difference in subchondral or epiphyseal trabecular bone thickness in medial vs. lateral
1	5-10% increase in subchondral or epiphyseal trabecular bone thickness in medial vs. lateral
2	11-25% increase in subchondral or epiphyseal trabecular bone thickness in medial vs. lateral
3	26-50% increase in subchondral or epiphyseal trabecular bone thickness in medial vs. lateral, obvious reduction in marrow spaces in outer ¾ of medial tibia
4	51-75% increase in subchondral or epiphyseal trabecular bone thickness in medial vs. lateral, generally has very little marrow space in outer ¾ of medial tibia, marrow spaces remain adjacent to cruciates
5	76-100% increase in subchondral or epiphyseal trabecular bone thickness in medial vs. lateral, generally has very little marrow space remains in medial tibia

### RNA preparation and analysis

Rabbit synovium was homogenized with 1 mm zirconia beads (Biospec Product Inc) in RNA STAT 60 (Tel-Test, Inc, Friendswood, TX, USA) or Trizol (Invitrogen) using Biospec homogenizer and total RNA was isolated according to manufacturer’s protocol. Trizol-treated samples were chloroform extracted [32,33] and processed using spin columns as instructed (SV total RNA isolation system; Promega, Madison, WI, USA). The cDNA was made using high-capacity cDNA RT kit (Applied Biosystems, Carlsbad, CA, USA). Rabbit transcript levels were determined by TaqMan RT-PCR using specific primers (Applied Biosystems) for catK (Oc03398667_m1), cysC (Oc03398476_m1) and MMP13 (Oc03396895_m1). All values were normalized by 18S rRNA (4333760F) expression. rAAV vector genomes in back-extracted DNA and vector-produced mouse cysC transcript levels in cDNA samples were measured by TaqMan RT-PCR using primers and probe specific to BGH polyA. Values were expressed by copies per 1 × 10^6^ cells (5 pg DNA/cell).

### Statistical analysis

*In vitro* data represented a minimum of two to three independent observations and were performed a minimum of two times. Data were analyzed for significance by unpaired Student’s *t* test. Histology scoring data were analyzed by Student’s *t* test and nonparametric Mann–Whitney *U* test for comparison between each group and sham control. For comparisons between treatment groups one-way analysis of variance (ANOVA) and nonparametric Kruskal-Wallis test were used. Analyses were performed using GraphPad Prism statistical software (GraphPad Software, Inc, La Jolla, CA, USA). *P* <0.05 was considered statistically significant.

## Results

### Cathepsin activity assay and inhibition by cysC *in vitro*

**Figure 1:**
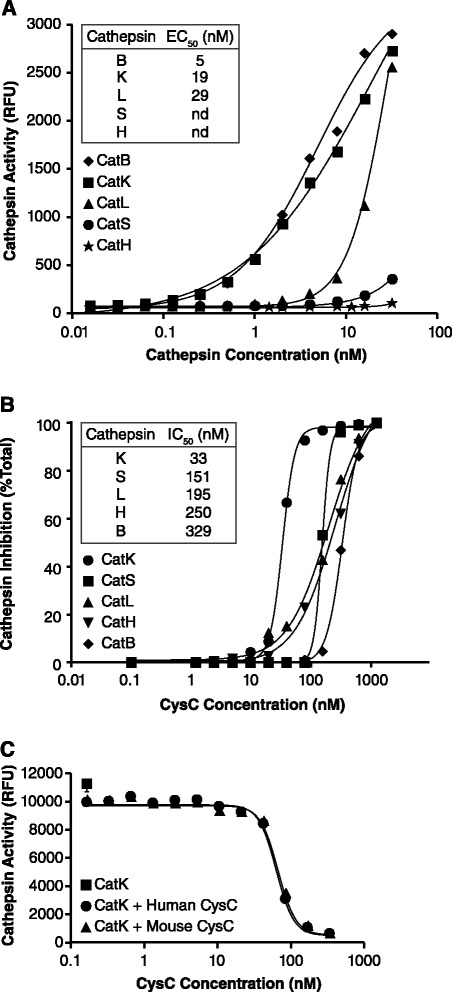
**Characterization of various cathepsins and their inhibition by cysC** ***in vitro*** **. (A)** Evaluation of cathepsins in activity assay. Cleavage of 10 μM substrate by 30 nM of each cathepsin (catB, catK, catL, catS and catH) at 30 min was measured by RFUs as described in Methods. Values represent means for each condition (n = 2). A representative of two independent experiments is shown. Insert shows EC_50_ for each cathepsin tested. Mean EC_50_ = 21 nM ± 4 for catK was based on four independent experiments. **(B)** Inhibition of cathepsin activity by cysC. Assays were performed using 25 nM of cathepsin and 50 μM of substrate in two to three independent experiments and the values represent means for each condition (n = 2–3). The data are shown as percentage of inhibition of activity compared no cysC present. Insert shows IC_50_ values at conditions used. **(C)** Inhibition of human catK by cysC. The potency of mouse and human cysC on catK activity (60 nM) with 50 μM of substrate was compared and inhibition was measured as RFUs at 20 min. IC_50_ was 65 nM for both cysC proteins. cat, cathepsin; cysC, cystatin C; RFU, relative fluorescence units.

Activity of various cathepsins reported to be present in the joint were tested. Similar to published reports [9,23,24], catK, catB and catL cleaved Z-Phe-Arg-AMC substrate (Figure 1A). Evaluation of their inhibition by cysteine protease inhibitor, cysC, in our conditions confirmed that cysC was a potent inhibitor of cathepsin activity (Figure 1B). K_i_ determinations by others have shown the tightest binding by catK, catL and catS to cysC [8,24]. Both human and mouse cysC proteins equally blocked human catK activity (Figure 1C). These results indicated that our cathepsin activity assay had the potential to detect presence of catK, catB and catL and they all could be inhibited by cysC protein.

### CatK production by rabbit synovial cells and inhibition by cysC

**Figure 2:**
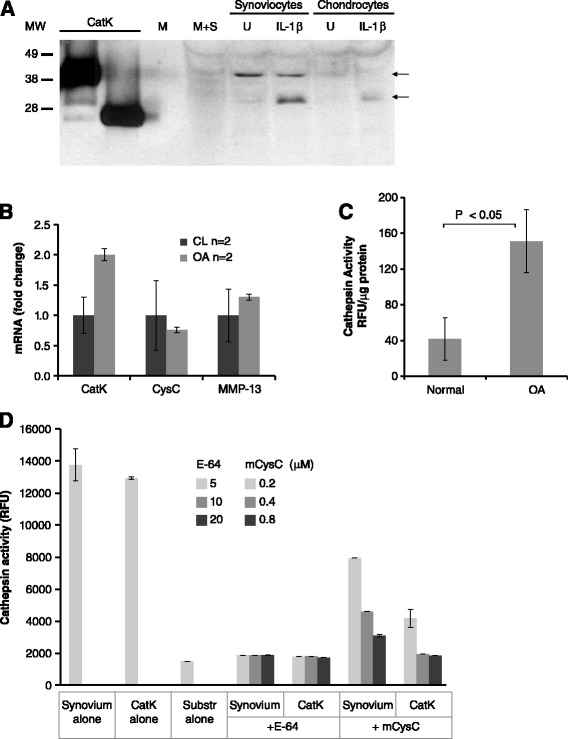
**Cathepsin expression by rabbit synovium and inhibition by cysC. (A)** Secretion of rabbit catK *in vitro*. Rabbit primary synoviocytes and chondrocytes were cultured in the absence (U = untreated) and presence of IL-1β (2 ng/ml). Culture media was analyzed for catK protein by Western blot. Purified human catK protein (proform and mature forms), media alone (M) and media with 10% serum (M + S) were run as controls (lanes 1–4 on the left). Protein molecular weight markers (MW) in kDa are shown on the left. Arrows indicate location of catK proform (35 kDa) and mature forms (27 kDa) in the culture media (right). **(B)** CatK expression in synovium in early rabbit OA. Synovium of normal (sham) and OA joints 4 weeks post ACLT were analyzed for mRNA levels by qPCR. CysC and MMP-13 expression are shown for comparison (n = 2/treatment). **(C)** Comparison of cathepsin activity in synovium of normal and rabbit OA (4 weeks) joints. Values represent the group mean ± standard deviation (SD) (n = 3) (three independent experiments). Significance by two-tailed Student’s *t* test. **(D)** Inhibition of cathepsin activity in rabbit synovium by cysC *in vitro*. Inhibitors, cysC (mouse) and E-64, were added into 4-week OA homogenates (‘synovium’) and incubated for 1 h followed by cathepsin activity assay. The effect of inhibitors on activity by equal amount of purified catK (50 nM) is shown for comparison. Values represent the group mean ± SD (n = 2). ACLT, anterior cruciate ligament transection; cat, cathepsin; cysC, cystatin C; E-64, trans-epoxysuccinyl-L-leucylamido-(4-guanidino)butane; IL-1β, interleukin 1 beta; kDa, kiloDaltons; MMP, matrix metalloproteinase; MW, molecular weight; OA, osteoarthritis.

CatK and other cathepsins are mostly localized to the lysosomal compartment in the cell. To test for catK secretion by rabbit joint cells, primary rabbit synoviocytes and chondrocytes were evaluated for catK levels in conditioned media. A 35 kiloDalton (kDa) proform and a 27 kDa mature form of catK were detected in the media from synoviocytes while lesser amounts were secreted by chondrocytes (Figure 2A). Inflammatory conditions such as treatment with IL-1β increased the secretion of mature form from both cell types. Thus, rabbit synoviocytes had the ability to secrete catK and could therefore have an extracellular role in OA pathology.

**Figure 3:**
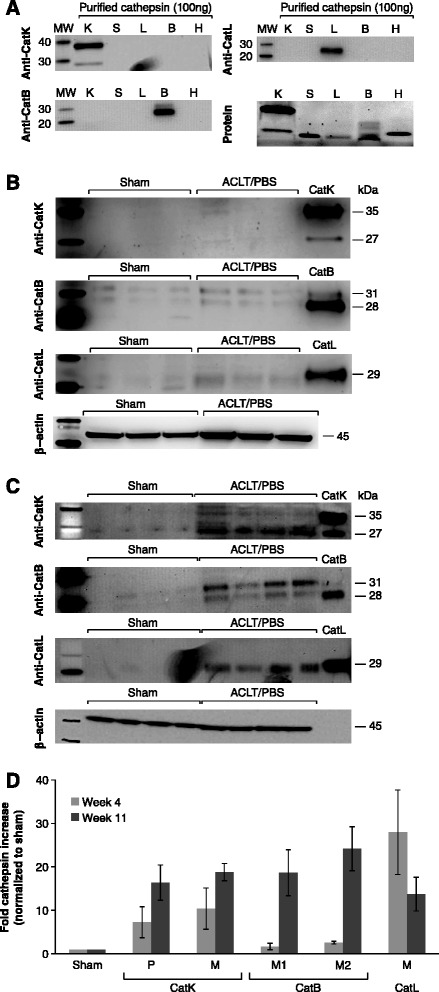
**Cathepsin K, B and L expression in rabbit synovial tissues** ***in vivo*** **. (A)**. Specificity of antibodies to catK, catB and catL was tested using purified proteins (100 ng/lane; equal loading was confirmed by Sypro protein staining) and showed no cross-reactivity. Cathepsin protein levels in synovium were tested 4 **(B)** and 11 weeks **(C)** after OA induction. Synovium samples (20 ug total protein) from sham and ACLT/PBS treatment groups were analyzed by Western blot (n = 3-4/group). Purified catK, catB and catL proteins (50 ng/lane) are shown as size markers. **(D)** Quantitation of cathepsin levels in synovium at 4 and 11 weeks post OA induction**.** Signal intensity was quantitated and the relative cathepsin increase in OA synovium compared to sham is shown as group mean ± standard deviation (SD) (n = 3-4/group). P, proform; M, mature form. ACLT, anterior cruciate ligament transection; cat, cathepsin; OA, osteoarthritis; PBS, phosphate-buffered saline.

We next evaluated catK expression and cathepsin activity in the synovium during early rabbit OA (4 weeks post ACLT). CatK mRNA levels were elevated twofold while no significant increase was observed for cysC or MMP-13 expression (Figure 2B). Furthermore, fourfold-increased cathepsin activity was measured in OA compared to normal (sham) synovium (Figure 2C). To evaluate whether OA-induced cathepsin activity could be inhibited by exogenously added cysC protein *in vitro*, mouse cysC protein was added to synovium lysates and significantly reduced activity was observed (Figure 2D). Comparable inhibition was obtained on activity of purified catK protein. Trans-epoxysuccinyl-L-leucylamido-(4-guanidino)butane (E-64), a small molecule inhibitor of cysteine protease, completely blocked activity of lysates confirming that the activity measured was due to cysteine proteases. Since the activity assay was not specific to catK, other cathepsins likely also contributed to the observed activity. To investigate this, we analyzed catK, catB and catL protein levels in normal and OA synovium (4 and 11 weeks post OA induction) (Figure 3). Low levels of catK and catB proteins were detected with higher increase observed for catL at 4 weeks. All three were clearly elevated 11 weeks post OA induction compared to sham animals. CatK was detected both as proform and mature form while catB (two mature forms, 28 and 31 kDa [6]) and catL were present as mature forms. Taken together, these results demonstrate that multiple cathepsins are elevated in rabbit OA synovium and that their activity can be inhibited by mouse cysC.

### rAAV transduction of rabbit joint cells *in vitro* and *in vivo*

**Figure 4:**
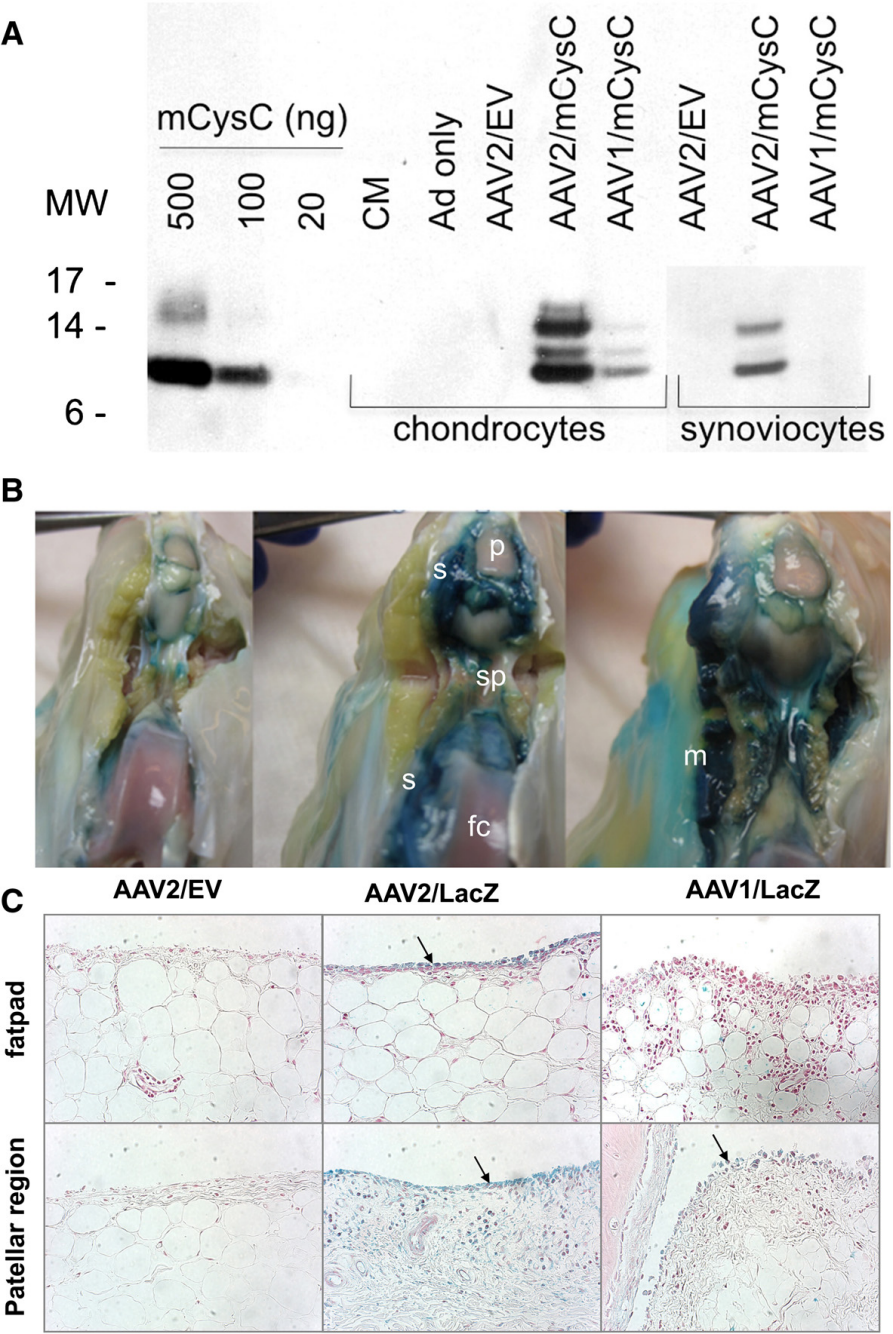
**Transduction of rabbit joint cells with rAAV1 and rAAV2 vectors** ***in vitro*** **and** ***in vivo*** **. (A)** *In vitro* transduction of rabbit primary synoviocytes and chondrocytes with rAAV1/mCysC and rAAV2/mCysC. Transductions with adenovirus ts149 helper virus alone (‘Ad only’) and AAV2/EV were run as negative controls. Culture media was analyzed by Western blot 48 h postinfection. Protein molecular weight markers (MW) and purified mcysC protein were run as controls. Multiple protein sizes likely represent different degrees of glycosylation. **(B-C)** AAV capsid serotype comparison and identification of transduced cell types in rabbit joints *in vivo*. rAAV2/EV, rAAV1/LacZ and rAAV2/LacZ were administered by IA route (n = 3/treatment) and vector transduction sites were located by staining for β-gal activity 4 weeks post delivery. Panel B. Macroscopic analysis of rabbit joints after β-gal staining. The distal portion of the patella ligament was transected transversely to create a flap that was lifted and dissected proximally toward the body to expose the front and sides of the joint. Abbreviations: s, synovium; p, patella; fc, femoral condyle; sp, suprapatella pouch; m, muscle. Panel C. Histological analysis of synovium for β-gal positive cells. Representative sections of synovium from fat pad and patellar region counterstained with NFR are shown. Arrows indicate localization of rAAV-transduced (blue) cells. Magnification x400. EV, empty vector; IA, intra-articular; mCysC, mouse cystatin C; MW, molecular weight; NFR, nuclear fast red; rAAV, recombinant adeno-associated virus.

rAAV1 and AAV2 vectors encoding cysC were generated and tested for cysC secretion by rabbit primary synoviocytes and chondrocytes *in vitro.* Analysis of culture media showed that both cell types secreted cysC with higher levels detected from rAAV2-treated cells compared to that of rAAV1 (Figure 4A).

To identify optimal capsid serotype for synovial transduction in rabbit joints *in vivo*, the rAAV1 and rAAV2 vectors encoding *LacZ* were administered into rabbit joints. Macroscopic and histological analyses for β-galactosidase activity showed that rAAV2 transduced rabbit synovium more efficiently than rAAV1, consistent with our *in vitro* analyses (Figure 4B, C). Robust rAAV2 transduction was detected in the synovial lining from the fat pad, patellar region, suprapatellar pouch and femur. Some β-galactosidase activity was detected in subsynovium adipocytes while little rAAV2 transduction was observed in chondrocytes, osteocytes, fibrocytes and skeletal myofibers (data not shown). In contrast, rAAV1 vector resulted in strong staining of surrounding muscle tissue with lighter staining detected in the synovium. Little staining was seen in the negative control (rAAV2/EV)-treated joints by histological analysis. Based on these results, AAV2 capsid was chosen for rAAV/cysC vector for synovial delivery.

### CysC gene transfer to synovium and effect on synovial cathepsin activity

**Figure 5:**
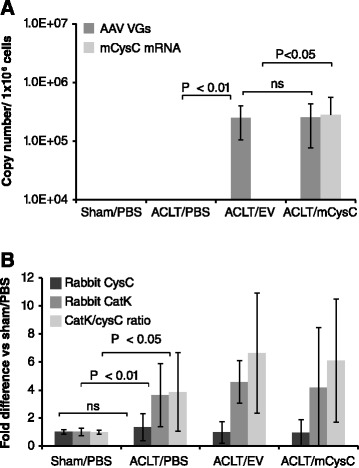
**rAAV2 mediated cysC gene transfer into rabbit joints.** rAAV vectors were administered 4 weeks post OA induction and the synovium was collected 7 weeks after vector injection. **(A)** Quantitation of rAAV vector genomes (VGs) and mCysC mRNA levels in the synovium. Detection of BGH polyA sequence by RT-PCR assay was expressed as copies per 1 x 10^6^ cells. **(B)** Analysis of rabbit catK and cysC mRNA levels in the synovium 11 weeks post ACLT. Values in treatment groups were expressed as fold increase over normal (sham/PBS). All values represent the group mean ± standard deviation (SD) (n = 7/group). Significance by two-tailed Student’s *t* test. ACLT, anterior cruciate ligament transection; BGH, bovine growth hormone; (m)CysC, (mouse) cystatin C; OA, osteoarthritis; PBS, phosphate-buffered saline; rAAV, recombinant adeno-associated virus.

To evaluate the effect of overexpression of cysC on cathepsin activity in the synovium, rAAV2/mCysC was delivered into rabbit OA joints. Seven weeks later, gene transfer was confirmed by detection of vector genomes in the synovium of rAAV2/EV and rAAV2/mCysC-treated joints (Figure 5A). Vector-derived cysC transcripts were also detected in rAAV2/mCysC-treated but not in rAAV2/EV-treated joints as expected. We then determined mRNA levels for endogenous rabbit catK and cysC in the synovium of all the treatment groups. Rabbit catK transcripts were increased fourfold in OA joints while there was no change in rabbit cysC mRNA levels compared to sham joints (Figure 5B). Thus, the ratio of rabbit catK to cysC expression was significantly increased in the synovium of all OA joints indicating a lack of coordinated upregulation. As expected, the viral cysC gene transfer did not alter endogenous rabbit catK or cysC mRNA expression.

**Figure 6:**
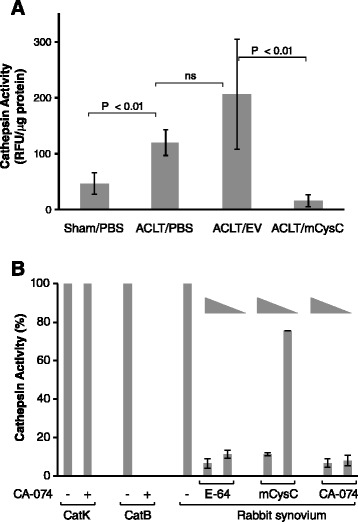
**Effect of cysC transfer on synovial cathepsin activity. (A)** Cathepsin activity in rabbit synovium at 11 weeks. Activity by RFU values from each treatment group was normalized by total protein content and represent the group mean ± standard deviation (SD) (n = 7/group). Significance by two-tailed Student’s *t* test. **(B)** Characterization of cathepsin activity in the rabbit synovium. ACLT/PBS lysates (n = 3 rabbits) were analyzed for activity in the presence of 0.5 and 0.1 μM (triangle) concentration of E-64, mCysC or CA-074 (catB-specific inhibitor). Purified catK and catB (50 nM each) proteins were tested for inhibition by CA-074 as controls. Data are shown as percentage of activity compared to no inhibitor. ACLT, anterior cruciate ligament transection; E-64, trans-epoxysuccinyl-L-leucylamido-(4-guanidino)butane; (m)CysC, (mouse) cystatin C; PBS, phosphate-buffered saline; RFU, relative fluorescence units.

We next examined cathepsin activity in the synovium to test whether cysC overexpression reduced this activity as expected if sufficient levels of functional cysC protein were produced from vector-infected cells. Similar to the fourfold increase in catK transcript levels, cathepsin activity was increased three- to fivefold in the OA synovium from PBS- and AAV2/EV-treated joints compared to sham joints (Figure 6A). In the rAAV2/mCysC-treated joints, however, there was a significant decrease in cathepsin activity. In fact, the virally delivered and *in vivo*-made cysC completely blocked synovial cathepsin activity and was comparable to that observed in sham-operated joints. Since our assay detected activities by catK, catB and catL and they were all elevated at week 11 (Figure 3C, D), the activity assays were repeated using a more catK-specific substrate (Z-GPR-AMC) or in the presence of catB-specific inhibitor (CA-074). These results showed that the majority of the activity detected was due to catB (Figure 6B) while the use of catK-specific substrate showed little activity (not shown). However, irrespective of the source of activity, all synovial cathepsin activity was reduced to that of normal synovium after cysC gene transfer.

### Effect of synovial cysC overexpression on synovium, cartilage and bone pathology

**Figure 7:**
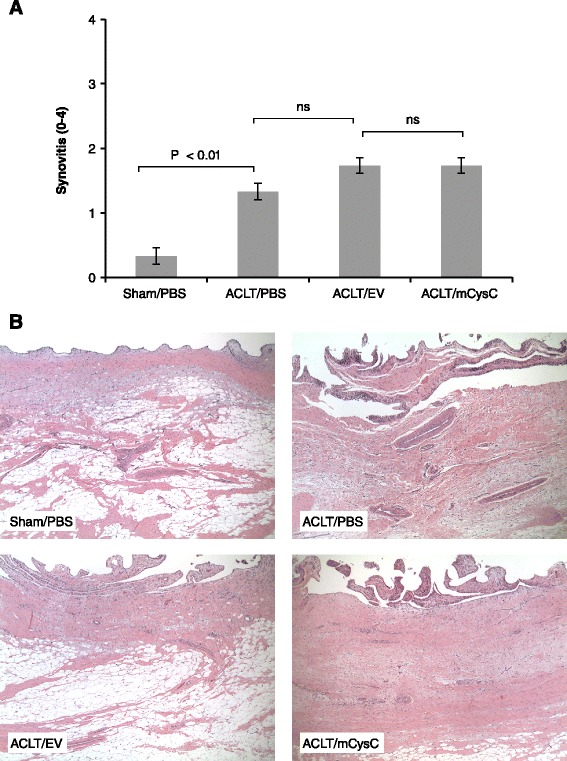
**Effect of synovial cathepsin inhibition on synovial pathology. (A)** Synovial pathology. H&E-stained sections were scored for inflammation as described in Methods. All values represent the group mean ± standard error (SE) (n = 15/treatment group). **(B)** Representative synovial histology for all treatment groups. The images shown are from synovium representing an average score of the group (magnification, x50). H&E, hematoxylin and eosin.

Evaluation of synovial morphology showed that all OA joints had significantly increased inflammation compared to sham joints (Figure 7A, B). There was no significant difference among PBS- or rAAV/EV-treated synovium indicating that rAAV delivery itself did not increase pathology. Furthermore, cysC overexpression did not alter the synovial inflammation score.

**Figure 8:**
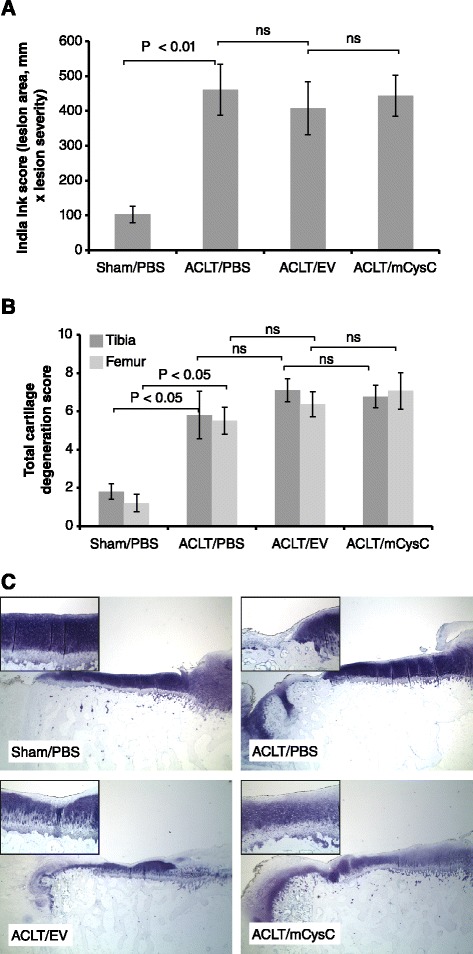
**Effect of synovial cathepsin inhibition on articular cartilage pathology. (A)** Cartilage surface evaluation by India ink staining. Values represent the group mean ± standard error (SE) (n = 15/treatment group). No difference was observed between ACLT-treatment groups. **(B)** Histological scoring of cartilage degradation. Scoring was performed using toluidine blue-stained sections. No difference was observed between ACLT groups while there was surgery-induced cartilage damage in all ACLT groups compared to their contralateral sham joints. All values represent the group mean ± SE (n = 15/treatment group). Methods for cartilage scoring and statistical analyses are described in Methods. **(C)** Representative cartilage histology for all treatment groups stained with toluidine blue. Images are from the medial side of tibial plateau and represent a cartilage section with an average score of the group (magnification, x16; insert, x100). ACLT, anterior cruciate ligament transection.

Cartilage degradation was examined both by gross assessment of tibial and femoral cartilage surfaces and by histological evaluation. Both methods showed that all ACLT-treated joints had significantly elevated levels of cartilage degradation compared to sham joints (Figure 8A,B,C). The OA pathology was most severe on the medial tibia and femur and consisted of cartilage lesions and chondrocyte hypertrophy detected in the majority of ACLT-treated animals. Delivery of rAAV2/mCysC or /EV vectors did not reduce any of these end points compared to PBS-treated OA joints.

Last, osteophyte formation and subchondral bone sclerosis were analyzed in tibial and femoral sections. Bone thickening was observed subjacent to cartilage lesions in ACLT-operated rabbits. Both osteophyte formation and bone sclerosis were significantly increased in OA joints compared to sham controls (not shown). However, no differences were observed in these scores between the ACLT treatment groups. Taken together of all the observations, overexpression of cysC in the synovium did not reduce synovitis, cartilage degradation or bone structure in established rabbit OA.

## Discussion

CatK levels are elevated in cartilage, bone and synovium during OA progression and recent efficacy with catK pharmacological inhibitors support targeting catK for prevention of cartilage loss [3,6-9,11-14,17-19]. Of the multiple proteases elevated during OA development, catK may be one of the principal proteases contributing to cartilage degradation in established OA as both the inflammatory and acidic conditions associated with increasing severity of OA augment catK activation [3,7,11,12,14]. While the pathological role of catK in cartilage and subchondral bone has gained support, the role of catK in synovium either in synovitis or as a secreted factor affecting cartilage pathology is unclear due to limited studies on inhibiting catK expression in the synovium. A recent rabbit OA study demonstrated that small interfering RNA (siRNA)-mediated gene silencing of catK in the synovial lining at the time of OA induction actually increased cartilage degradation indicating an unexpected protective role of catK in the synovium [20]. To explore the hypothesis that catK and other cathepsins expressed in the synovium have a significant role in OA pathology and to model a more clinically relevant setting, we inhibited the function of cathepsins in the face of preexisting OA. This was achieved by overexpression of their natural inhibitor, cysC, which blocks the activity of cathepsins by forming a reversible enzyme-inhibitor complex [8]. Other cathepsins elevated in OA synovium, such as catB and catL, and likely contributing to OA pathology can also be inhibited by this approach [4,5,10,23,24]. Our results interestingly do not support the role of increased levels of catK, catB or catL in the synovium in OA progression.

We used rAAV vector-mediated gene delivery of cysC to provide local production of the inhibitor. Intra-articular delivery of the vector transduced mostly synovium thereby facilitating the investigation of the role of cathepsins at this site. Our data and published reports indicated no change in endogenous cysC levels unlike increased catK expression in synovium during OA progression [4,12,13]. Delivery of cysC gene increased the inhibitor production *in vivo* and restored the normal cathepsin-cysC balance in the synovium as indicated by the normalized synovial cathepsin activity (this represented total cathepsin activity since our assay did not distinguish between intra- and extracellular location of cathepsins). Although cysC exhibits a higher affinity for catK than catB [8], the use of catB-specific inhibitor CA-074 revealed that the majority of the detected cathepsin activity in OA synovium was due to catB. Our inability to detect catK activity despite significantly increased catK transcript and protein levels may have been due to instability of active catK or interference by high levels of catB [8]. In addition to catK, both catB and catL were increased in rabbit OA synovium similar to reports in human arthritic synovium and are thought to contribute to cartilage degradation [4,5,34]. Therefore, inhibition by cysC allowed us to examine the overall contribution of these synovial cathepsins to OA pathology.

Our data show, for the first time, that blocking cathepsin activity by overexpression of cysC in the synovium had no effect on OA cartilage pathology in established OA. However, systemic delivery of pharmacological catK inhibitors have shown reduced OA cartilage degradation especially when used as a prophylactic treatment or shortly after disease induction [17-19]. This indicates that early intervention is likely critical to obtain efficacy. Another explanation may be the delivery route of the therapeutic agent as small molecule catK inhibitors administered systemically can access areas such as subchondral bone that would be limited by intra-articular delivery of rAAV vector. Thus, catK activity in subchondral bone and bone marrow may play a significant pathological role for cartilage loss and is supported by recent reports on changes in osteoarthritic subchondral bone [35-37]. Lastly, it is possible that little cathepsins are secreted into extracellular matrix in cartilage *in vivo*. As the majority of cathepsins are localized intracellularly, the extracellular location of secreted cysC would be expected to have little access to their activity and hence have no effect on any degradation of collagen taken up by phagocytosis [38]. This is in contrast to small synthetic cathepsin inhibitors that can access lysosomes and hence inhibit intracellular cathepsin activity therefore possibly explaining their protective effect on cartilage.

Similarly, overexpression of cysC in the synovium had no effect on synovial inflammation in our rabbit OA model. Yet, we observed elevated levels of catK expression and upregulation of catK expression in a transgenic mouse model was shown to increase synovitis [15]. Though our *in vitro* data and others have shown cathepsin secretion during inflammatory conditions present in rheumatoid synovium [6-8,10], secretion of cathepsins may occur less in OA synovium. In this case, little or no contact of extracellular cysC protein with the intracellular cathepsins could explain the lack of effect by cysC. Increased catK expression in the synovium is likely caused by low level of inflammation occurring in a preexisting disease. This is supported by elevation of catK levels after IL-1β stimulation in our *in vitro* studies and by reports by others in multiple chronic inflammatory conditions *in vivo* [7,10,39]. Lastly, we observed no changes in osteophyte formation or bone sclerosis by cysC overexpression in the synovium despite reports on the ability of cysC to stimulate bone formation [40].

Potential limitations in our studies were the use of a single animal model and one time point for histological analyses. However, the rabbit ACLT model is a well-established animal model of OA and has been used for studying the role of catK in a number of previous studies [12,18,20,29,30,41]. The time point used was chosen based on rAAV-mediated cysC production starting likely around 2 to 3 weeks post delivery and therefore exposing the synovium for continuous cysC levels for about 7 weeks. Previously, it was demonstrated that 4 weeks was sufficient to observe cartilage repair after intra-articular delivery of rAAV2 expressing basic fibroblast growth factor (bFGF) and the repair was more prominent after 8 weeks [41]. Others have demonstrated efficacy with catK inhibitors with systemic delivery during early stages of disease [17-19]. We did not attempt the pretreatment model to understand the role of catK in preexisting disease that is more relevant for identification of therapeutic targets and for predicting clinical utility.

## Conclusions

We have shown elevated catK expression and increased production of catK, catB and L proteins in rabbit synovium during OA. Synovial cathepsin activity was blocked by overexpression of cysC in the synovium by rAAV2 vector-mediated delivery demonstrating both the feasibility of a robust gene delivery for a therapeutic agent and a production of biologically active protein in a large animal model. However, the inability of overexpressed cysC to reduce cartilage degradation or synovitis suggests little effect by secreted cathepsins during ongoing OA and highlights the importance of appropriate location of the therapeutic agent during the treatment.

## Abbreviations

ACLT: anterior cruciate ligament transection
bFGF: basic fibroblast growth factor
BGH: bovine growth hormone
catK: cathepsin K
CMV: cytomegalovirus
cysC: cystatin C
drp: DNAse resistant particles
E-64: trans-epoxysuccinyl-L-leucylamido-(4-guanidino)butane
EV: empty vector
FBS: fetal bovine serum
H&E: hematoxylin and eosin
HRP: horseradish peroxidase
IgG: immunoglobulin G
IL-1β: interleukin 1 beta
kDa: kiloDaltons
KO: knockout
MMP: matrix metalloproteinase
MW: molecular weight
NBF: normal buffered formalin
NFR: nuclear fast red
OA: osteoarthritis
PBS: phosphate-buffered saline
PFA: paraformaldehyde
rAAV: recombinant adeno-associated virus
RFU: relative fluorescence units
RT-PCR: real-time PCR
siRNA: small interfering RNA
